# Acute Impact of Hourly Ambient Air Pollution on Preterm Birth

**DOI:** 10.1289/EHP200

**Published:** 2016-04-29

**Authors:** Shanshan Li, Yuming Guo, Gail Williams

**Affiliations:** School of Public Health, The University of Queensland, Brisbane, Queensland, Australia

## Abstract

**Background:**

Preterm birth is a major perinatal health problem, but factors leading to it are still not completely understood.

**Objectives:**

Our goal was to identify the relation between acute increase in ambient air pollution in a few hours before onset of labor and the risk of preterm birth.

**Methods:**

We collected registered birth outcome data and hourly ambient air pollution measurements during 2009‒2013 in Brisbane, Australia. Using a time-stratified case-crossover design and conditional logistic regression models with natural cubic splines, we assessed the shape of air pollution-preterm birth curve, after controlling for potential confounders. We also examined the effect modification of other factors.

**Results:**

The association between air pollution [nitrogen dioxide (NO2), sulfur dioxide (SO2), and carbon monoxide (CO)] and preterm birth was nonlinear. Threshold concentrations for the mean of 0‒24 hr NO2, 24‒48 hr SO2, and 24‒48 hr CO before onset of labor were 7.6 parts per billion (ppb), 3.8 ppb, and 162.5 ppb, respectively. Increases in air pollution concentrations above thresholds were associated with increased risks of preterm birth. The odds ratios of preterm birth at the 95th percentile of NO2, SO2, and CO against the thresholds were 1.17 (95% CI: 1.08, 1.27), 1.01 (95% CI: 0.99, 1.04), and 1.18 (95% CI: 1.06, 1.32), respectively. The associations were modified by demographic factors, such as maternal smoking and socioeconomic status.

**Conclusion:**

Acute increases in ambient air pollution concentrations above certain levels before onset of labor may stimulate preterm birth.

**Citation:**

Li S, Guo Y, Williams G. 2016. Acute impact of hourly ambient air pollution on preterm birth. Environ Health Perspect 124:1623–1629; http://dx.doi.org/10.1289/EHP200

## Introduction

Preterm birth is a major perinatal health problem associated with neonatal mortality and morbidity and can cause long-term adverse health consequences in life (Beck et al. 2010; Lumley 2003). It was estimated that approximately 11% of all live births were born preterm worldwide in 2010, and this high and still rising incidence represents significant financial implications for health care systems (Blencowe et al. 2012). Yet factors leading to preterm birth are still not completely understood. In recent years, there has been a growing concern about the possible influence of air pollution on preterm birth. Most studies (Bobak 2000; Gehring et al. 2011; Hansen et al. 2006; Huynh et al. 2006; Jalaludin et al. 2007; Leem et al. 2006; Llop et al. 2010; Ritz et al. 2000, 2007; Suh et al. 2008, 2009; Wu et al. 2009) that investigated this relationship focused on exposures to air pollutants during the entire pregnancy or during specific trimesters, and the results were mixed. We have found no published studies on the impact of maternal exposure to air pollution in the few hours before onset of labor on the risk of preterm birth.

The aim of the present study was to identify the relation between maternal exposures to ambient air pollutants a few hours before onset of labor and preterm birth in Brisbane, Australia, from 2009 through 2013. We hypothesized that a short-term increase in ambient air pollution closely before onset of labor contributes to the risk of preterm birth and that this effect would be modified by demographic factors such as maternal age, maternal existing medical conditions, number of previous pregnancies, smoking status, multiple birth, infant sex, and socioeconomic levels.

## Materials and Methods

### Birth Information

We collected birth outcome data from the Queensland Health Perinatal Data Collection Unit for births during 1 January 2009‒31 December 2013 in the Brisbane metropolitan area, Australia. This registered database covers births from all public and private hospitals and voluntarily reported homebirths in Brisbane. Information used in this study includes birth status (live born or stillbirth), gestational age in weeks, date and time of birth, method of birth, length of labor, number of births, infant sex, maternal age, prepregnancy medical conditions, number of previous pregnancies, maternal smoking status, and an index of socioeconomic status (SES) linked to the living area during pregnancy. The definition of preterm birth is birth before a gestational age of 37 weeks (Goldenberg et al. 2008). Thus, we retained data on all live births occurring < 37 weeks of gestation in this study. The study was approved by the University of Queensland Medical Research Ethics Committee.

### Air Pollution and Meteorological Data

Hourly data on particulate matter ≤ 10 μm in diameter (PM_10_), particulate matter ≤ 2.5 μm in diameter (PM_2.5_), nitrogen dioxide (NO_2_), sulfur dioxide (SO_2_), ozone (O_3_), carbon monoxide (CO), ambient temperature, and relative humidity were obtained from the Queensland Government Department of Environment and Heritage Protection for five monitoring sites across Brisbane. PM_2.5_ and PM_10_ were measured by either a high- or low-volume air sampler or a tapered element oscillating microbalance (TEOM). NO_2_ was measured with chemiluminescence, which is a chemical reaction that emits energy in the form of light. SO_2_ was measured by a differential optical absorbance spectroscopy (DOAS) instrument. O_3_ concentrations were monitored through the principle of absorption of ultraviolet (UV) light. CO was measured by gas filter correlation.

For each preterm birth, we defined the onset of labor time as the birth time by a deduction of labor length. Then we calculated mean values of air pollutants and meteorological conditions by hourly measurements for the periods of 0‒24 hr, 24‒48 hr, 48‒72 hr, 0‒48 hr, and 0‒72 hr, respectively, before the time of labor onset if at least 75% of measurements were available in the corresponding period. Otherwise the data were considered missing.

### Statistical Analysis

We employed a time-stratified case-crossover design to examine the association between air pollution and preterm birth. The case-crossover design can be explained as a self-matched case–control study (Janes et al. 2005). For each individual, exposure information (e.g., air pollution) is collected for the “case” period (that is, the onset of labor time of preterm birth in this study) and a series of “control” periods that are not associated with the event of interest. In the time-stratified design, control periods should be selected from the fixed time strata (e.g., month) to avoid any “overlap bias” (Lumley and Levy 2000). In this study, we used the calendar month as the fixed time stratum, and control periods comprised the same hour of the same day of the week in the calendar month of preterm birth labor onset, to control for the effect of day of the week and intra-day variation. Air pollution and confounding information were obtained for the hour of the onset of labor event for both case and control periods.

We used conditional logistic regression models to fit the time-stratified case-crossover design, which successfully controlled for time-invariant individual level confounders (e.g., infant sex and maternal age), because comparisons between case and control periods were made within individuals. Mean values of the air pollutants (PM_10_, PM_2.5_, NO_2_, SO_2_, O_3_, and CO) for the periods of 0‒24 hr, 24‒48 hr, 48‒72 hr, 0‒48 hr, and 0‒72 hr preceding the time of labor onset were examined separately in single-pollutant models. To fully adjust for the potential time-variant confounders, we used natural cubic splines with 4 degrees of freedom (df) for the 0‒72 hr mean values of ambient temperature and relative humidity in all models.

Natural cubic splines were also applied for air pollutants in single-pollutant models to check whether the associations between air pollutants and preterm birth were linear or nonlinear. We selected the df and the time frame of exposure before onset of labor by judging the model fit which is reflected by the Akaike Information Criterion (AIC). For each air pollutant, model with the lowest AIC value indicated the best df and exposure time frame. In case of linear relationship between air pollution and preterm birth, we calculated the odds ratios (OR) and the 95% confidence intervals (CI) of preterm birth at 75th and 95th percentiles of air pollution against the median concentration of air pollution. Otherwise, in case of nonlinear relationship, we calculated the OR and 95% CI of preterm birth at 75th and 95th percentiles of air pollution against the minimum preterm birth concentration of air pollution (threshold).

To determine the threshold of air pollutant, we first plotted the graph of the relationship between air pollutant and preterm birth, and then visually checked the possible range of the threshold. Afterward, we iteratively estimated the AIC values for conditional logistic regression models by 0.1-unit increments in air pollutant within the identified range of threshold from visual inspection using the segment spline model. The concentrations of air pollutants corresponding to the lowest AIC values were chosen as the thresholds (minimum preterm birth concentrations of air pollutants). This method has been widely used to test the threshold for nonlinear temperature effect on mortality (Chung et al. 2009; Kim et al. 2006; Yu et al. 2010).

To evaluate the potential confounding effects of other air pollutants on the association between an air pollutant and preterm birth, we ran two-pollutant and multiple-pollutant models, and compared the OR and 95% CI of preterm birth associated with a pollutant from the single-pollutant model with those from the two-pollutant or multiple-pollutant model. To assess the possible modification effects of demographic factors on the air pollution–preterm birth association, we conducted stratification analyses for different groups: maternal age (< 35 years vs. ≥ 35 years), prepregnancy medical conditions (yes vs. no), number of previous pregnancies (0 vs. ≥ 1), maternal smoking status [smokers (self-reported any smoking during pregnancy) vs. nonsmokers (self-reported no smoking during pregnancy)], number of births (single birth vs. multiple births), infant sex (girls vs. boys), and SES index [1‒5 (indicating low level) vs. 6‒10 (indicating high level)]. The statistical significance of difference between effect estimates for the above subgroups (e.g., maternal age < 35 years vs. ≥ 35 years), was examined by


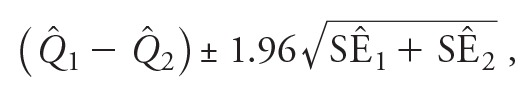
,

where *Qˆ*_1_ and *Qˆ*_2_ are the effect estimates for the two categories (e.g., < 35 years and ≥ 35 years), and SÊ_1_ and SÊ_2_ are their respective standard errors (Kan et al. 2008; Zeka et al. 2006). Planned cesarean sections were excluded in the analyses.

We added temperature variability (standard deviation of 0‒72 hr temperatures) to the models, to check whether the effects of air pollutants on preterm birth would be changed or not, because studies have reported that large temperature change increased risk of health events (Guo et al. 2011; Li et al. 2014; Qiu et al. 2013). We used 0‒12 hr average concentrations of air pollutants as exposure, to check whether the model fit was improved or not. All analyses were performed using R software (version 3.1.3) (R Core Team 2015). R codes were provided to show how to match case and control by the same hour of the same day of the week in the same month (see Supplemental Material, “R codes”).

## Results

Table 1 shows the maternal and fetal demographic characteristics of preterm births delivered in Brisbane, Australia from 1 January 2009 to 31 December 2013. A total of 6,949 preterm births occurred over the entire study period. Mothers < 35 years of age, without existing prepregnancy medical condition, ever got pregnant before the current pregnancy, never smoked, and mothers living in the area with high SES accounted for greater proportion of births. A majority of births were singletons, and the infant sexes were distributed evenly.

**Table 1:** Characteristics of mothers and preterm births in Brisbane, Australia, 2009–2013.

Variables	*n *(%)
Total	6949 (100.0)
Maternal age (years)
< 35	5260 (75.7)
≥ 35	1689 (24.3)
Prepregnancy medical conditions
No	5122 (73.7)
Yes	1827 (26.3)
Previous pregnancy
0	2514 (36.2)
≥ 1	4435 (63.8)
Maternal smoking status
Smokers	773 (11.1)
Nonsmokers	6176 (88.9)
Number of births
Single	4929 (70.9)
Multiple	2020 (29.1)
Infant sex
Female	3276 (47.1)
Male	3673 (52.9)
SES index
Index 1–5 (low level)	910 (13.1)
Index 6–10 (high level)	6039 (86.9)

Table 2 displays the hourly air pollution and meteorological exposure information during the study period. Mean levels of hourly PM_2.5_, PM_10_, NO_2_, SO_2_, O_3_, CO, ambient temperature, and relative humidity were 6.32 μg/m^3^, 17.27 μg/m^3^, 6.52 ppb, 1.95 ppb, 17.27 ppb, 219.30 ppb, 21.97°C, and 70.82%, respectively. The summary statistics for daily air pollution and meteorological factors are similar to the hourly data (see Table S1). Hourly values of air pollutants and weather conditions were positively correlated with each other (see Table S2).

**Table 2:** Summary statistics of hourly air pollution and weather conditions during 2009–2013 in Brisbane, Australia.

Variables	Mean ± SD	Percentile				
		5th	25th	50th	75th	95th
PM_2.5_ (μg/m^3^)	6.32 ± 4.05	3.20	4.35	5.55	7.24	11.10
PM_10_ (μg/m^3^)	17.27 ± 15.33	9.78	13.08	15.79	18.94	26.29
NO_2_ (ppb)	6.52 ± 3.50	2.47	4.00	5.58	8.25	13.63
SO_2_ (ppb)	1.95 ± 3.26	0.42	0.75	1.15	2.00	5.36
O_3_ (ppb)	17.27 ± 8.59	5.33	10.56	16.11	23.00	32.89
CO (ppb)	219.30 ± 135.53	50.00	120.00	200.00	300.00	450.00
Temperature (°C)	21.97 ± 4.83	13.25	18.78	22.48	25.46	29.24
Relative humidity (%)	70.82 ± 14.31	44.99	60.66	73.18	82.34	90.16
Abbreviations: CO, carbon monoxide; NO_2_, nitrogen dioxide; O_3_, ozone; PM_2.5_, particulate matter ≤ 2.5 μm in diameter; PM_10_, particulate matter ≤ 10 μm in diameter; ppb, parts per billion; SD, standard deviation; SO_2_, sulfur dioxide.						

Our preliminary analyses (see Figure S1) showed that the associations between air pollutants and preterm birth were generally U-shaped for NO_2_, SO_2_, and CO—that is, the threshold effect (a minimum preterm birth concentration of the air pollutant) was indicated. There were no statistically significant relationships among PM_2.5_, PM_10_, and O_3_ and preterm births in single-pollutant models. Natural cubic splines with 3 df for the mean values of 0‒24 hr NO_2_, 24‒48 hr SO_2_, and 24‒48 hr CO preceding the time of labor onset, respectively, produced the best model fits for each air pollutant. Thus, we chose these three air pollutants with certain exposure time frames to estimate the risk of preterm birth at the 75th and 95th percentiles of pollutant against the threshold in all following analyses.

**Figure 1:**
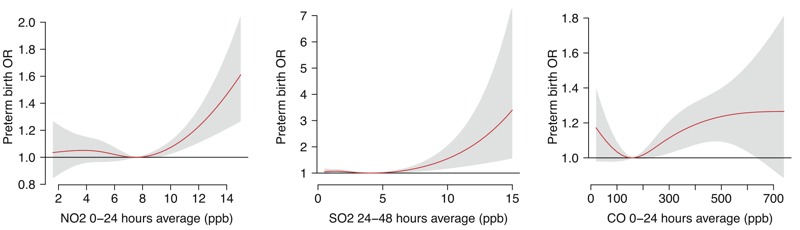
The relationships between air pollutants and preterm birth in single-pollutant models with 3 degrees of freedom natural cubic splines for air pollutants. Abbreviations: CO, carbon monoxide; NO_2_, nitrogen dioxide; OR, odds ratio; ppb, parts per billion; SO_2_, sulfur dioxide.

The threshold concentrations as listed in Table 3 were 7.6 ppb for the mean of antepartum 0‒24 hr NO_2_, 3.8 ppb for the mean of antepartum 24‒48 hr SO_2_, and 162.5 ppb for the mean of antepartum 24‒48 hr CO, respectively. Figure 1 clearly presents the U-shaped relationships between air pollutants and preterm birth. Increased concentrations of NO_2_, SO_2_, and CO above thresholds shortly before onset of labor were positively associated with increased risks of preterm birth. ORs for preterm birth at the 75th percentile of NO_2_, SO_2_, and CO against the thresholds were 1.01 (95% CI: 0.99, 1.03), 1.04 (95% CI: 0.99, 1.08), and 1.10 (95% CI: 1.01, 1.19), respectively; ORs for preterm birth at the 95th percentile of NO_2_, SO_2_, and CO against the thresholds were 1.17 (95% CI: 1.08, 1.27), 1.01 (95% CI: 0.99, 1.04), and 1.18 (95% CI: 1.06, 1.32), respectively (Table 3).

**Table 3:** The risks of preterm birth at 75th and 95th percentiles of air pollution against the minimum preterm birth concentration of air pollution (threshold) in single-pollutant models.

Pollutant	Threshold (ppb)	75th (ppb)	95th (ppb)	OR (95% CI)	
				75th vs. threshold	95th vs. threshold
NO_2_	7.6	8.3	11.3	1.01 (0.99, 1.03)	1.17 (1.08, 1.27)
SO_2_	3.8	2.2	4.8	1.04 (0.99, 1.08)	1.01 (0.99, 1.04)
CO	162.5	301.0	424.0	1.10 (1.01, 1.19)	1.18 (1.06, 1.32)
Abbreviations: CI, confidence interval; CO, carbon monoxide (mean of 0–24 hr preceding onset of labor); NO_2_, nitrogen dioxide (mean of 0–24 hr preceding onset of labor); OR, odds ratio; ppb, parts per billion; SO_2_, sulfur dioxide (mean of 24–48 hr preceding onset of labor).					

**Figure 2:**
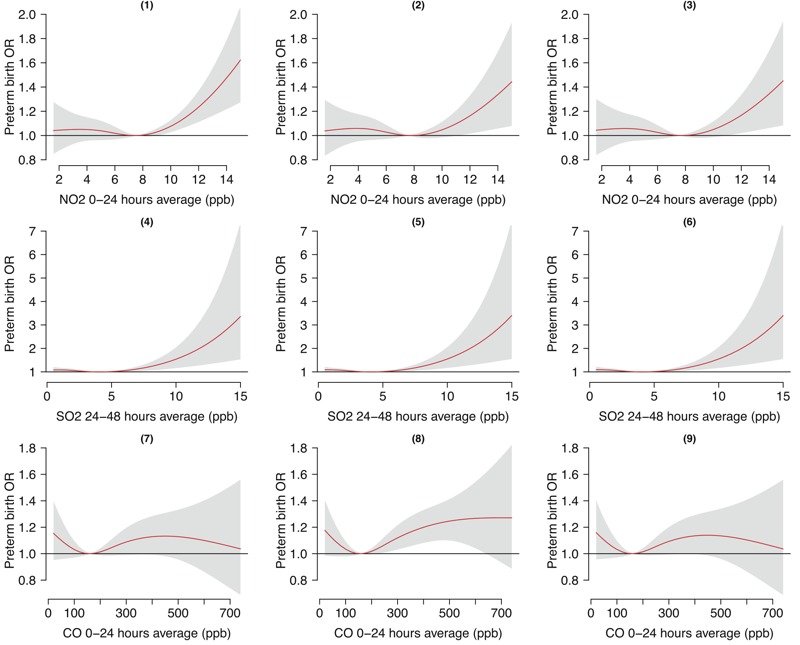
The relationships between air pollutants and preterm birth in two-pollutant and three-pollutant models with 3 degrees of freedom natural cubic splines for air pollutants. (1,4) NO_2_ + SO_2_; (2,7) NO_2_ + CO; (5,8) SO_2_ + CO; (3,6,9) NO_2_ + SO_2_ + CO. Abbreviations: CO, carbon monoxide; NO_2_, nitrogen dioxide; OR, odds ratio; ppb, parts per billion; SO_2_, sulfur dioxide.

The effect of each pollutant on the risk of preterm birth appeared independent of the other pollutants (Figure 2). The OR values of preterm birth associated with individual air pollutants were similar in two-pollutant and three-pollutant models compared with those in single-pollutant models (Table 4).

**Table 4:** The risks of preterm birth at 75th and 95th percentiles of air pollution against the minimum preterm birth concentration of air pollution (threshold) in two-pollutant and three-pollutant models.

Model	OR (95% CI)	
	75th vs. threshold	95th vs. threshold
NO_2_
NO_2_ + SO_2_	1.01 (0.99, 1.03)	1.17 (1.08, 1.27)
NO_2_ + CO	1.00 (0.98, 1.02)	1.13 (1.01, 1.26)
NO_2_ + SO_2_ + CO	1.00 (0.98, 1.03)	1.13 (1.01, 1.26)
SO_2_
NO_2_ + SO_2_	1.04 (0.99, 1.09)	1.01 (0.99, 1.04)
SO_2_ + CO	1.04 (0.99, 1.09)	1.01 (0.99, 1.04)
NO_2_ + SO_2_ + CO	1.04 (0.99, 1.09)	1.01 (0.99, 1.04)
CO
NO_2_ + CO	1.07 (0.97, 1.18)	1.11 (0.96, 1.27)
SO_2_ + CO	1.10 (1.01, 1.19)	1.18 (1.06, 1.32)
NO_2_ + SO_2_ + CO	1.07 (0.97, 1.18)	1.11 (0.96, 1.27)
Abbreviations: CI, confidence interval; CO, carbon monoxide (mean of 0–24 hr preceding onset of labor); NO_2_, nitrogen dioxide (mean of 0–24 hr preceding onset of labor); OR, odds ratio; ppb, parts per billion; SO_2_, sulfur dioxide (mean of 24–48 hr preceding onset of labor).		

Table 5 shows the results from stratification analyses for different groups evaluating whether demographic factors modified the air pollution–preterm birth relationship. There was no consistent increasing or decreasing trend of the risk within categories of maternal age and prepregnancy medical conditions. The associations of NO_2_ and CO with preterm birth were slightly stronger among those who had ever got pregnant, had multiple births, and had female babies. The risks of preterm birth associated with all three air pollutants were greater among smokers and families living in lower-SES areas. However, only smoking during pregnancy and low SES significantly modified the effects of NO_2_ and CO on preterm birth, respectively (see Table S3).

**Table 5:** Risks of preterm birth at 75th and 95th percentiles of air pollution against the minimum preterm birth concentration of air pollution (threshold) by level of demographic factors.

Factors	NO_2_ [OR (95% CI)]	SO_2_ [OR (95% CI)]	CO [OR (95% CI)]
Maternal age (years)
< 35
75th vs. threshold	1.00 (0.99, 1.01)	1.06 (0.95, 1.17)	1.12 (0.94, 1.33)
95th vs. threshold	1.26 (1.09, 1.47)	1.01 (0.97, 1.05)	1.30 (1.05, 1.61)
≥ 35
75th vs. threshold	1.01 (0.97, 1.06)	1.03 (0.98, 1.08)	1.09 (0.99, 1.20)
95th vs. threshold	1.14 (1.03, 1.27)	1.02 (0.99, 1.05)	1.14 (1.01, 1.30)
Prepregnancy medical conditions
No
75th vs. threshold	1.01 (0.99, 1.02)	1.04 (0.99, 1.10)	1.12 (1.02, 1.24)
95th vs. threshold	1.18 (1.07, 1.30)	1.01 (0.99, 1.03)	1.24 (1.09, 1.41)
Yes
75th vs. threshold	1.67 (1.07, 2.61)	1.02 (0.95, 1.10)	1.11 (0.47, 2.59)
95th vs. threshold	1.90 (1.18, 3.04)	1.03 (0.97, 1.09)	1.11 (0.51, 2.40)
Previous pregnancy
0
75th vs. threshold	1.00 (0.99, 1.01)	1.10 (1.00, 1.22)	1.02 (0.93, 1.13)
95th vs. threshold	1.06 (0.93, 1.21)	1.00 (0.99, 1.01)	1.09 (0.93, 1.26)
≥ 1
75th vs. threshold	1.01 (0.99, 1.04)	1.01 (0.97, 1.05)	1.15 (1.03, 1.29)
95th vs. threshold	1.24 (1.12, 1.37)	1.03 (0.99, 1.08)	1.25 (1.09, 1.44)
Smoking
Yes
75th vs. threshold	2.02 (1.05, 3.89)	1.08 (0.95, 1.24)	2.35 (0.58, 9.63)
95th vs. threshold	2.59 (1.28, 5.25)	1.04 (0.97, 1.11)	2.47 (0.69, 8.82)
No
75th vs. threshold	1.01 (0.99, 1.03)	1.03 (0.98, 1.08)	1.06 (0.98, 1.15)
95th vs. threshold	1.16 (1.06, 1.27)	1.01 (0.99, 1.04)	1.15 (1.03, 1.28)
Number of births
Single
75th vs. threshold	1.00 (0.99, 1.01)	1.07 (0.99, 1.15)	1.09 (0.98, 1.21)
95th vs. threshold	1.12 (1.02, 1.23)	1.00 (1.00, 1.00)	1.14 (1.00, 1.30)
Multiple
75th vs. threshold	1.03 (0.96, 1.10)	1.01 (0.97, 1.04)	1.11 (0.95, 1.28)
95th vs. threshold	1.32 (1.12, 1.55)	1.10 (1.01, 1.19)	1.28 (1.06, 1.55)
Infant sex
Female
75th vs. threshold	1.01 (0.98, 1.05)	1.01 (0.96, 1.05)	1.16 (1.00, 1.34)
95th vs. threshold	1.20 (1.06, 1.36)	1.02 (0.97, 1.08)	1.26 (1.06, 1.49)
Male
75th vs. threshold	1.00 (0.99, 1.02)	1.07 (1.00, 1.14)	1.06 (0.96, 1.17)
95th vs. threshold	1.15 (1.03, 1.28)	1.01 (0.99, 1.04)	1.14 (1.00, 1.30)
SES index
Index 1–5
75th vs. threshold	1.02 (0.99, 1.05)	1.05 (0.95, 1.17)	3.25 (1.72, 6.15)
95th vs. threshold	1.19 (0.96, 1.46)	1.05 (0.98, 1.13)	3.27 (1.70, 6.27)
Index 6–10
75th vs. threshold	1.01 (0.99, 1.04)	1.03 (0.98, 1.09)	1.08 (0.99, 1.17)
95th vs. threshold	1.18 (1.07, 1.29)	1.01 (0.99, 1.03)	1.17 (1.05, 1.30)
Abbreviations: CI, confidence interval; CO, carbon monoxide (mean of 0–24 hr preceding onset of labor); NO_2_, nitrogen dioxide (mean of 0–24 hr preceding onset of labor); OR, odds ratio; SO_2_, sulfur dioxide (mean of 24–48 hr preceding onset of labor). *p*-Values for differences are presented in Table S3.			

The effects of air pollutants on preterm birth did not change when we put temperature variability to the models (see Figure S2). When we used 0‒12 hr average concentrations of air pollutants, the model fit was not improved.

## Discussion

In this study, we found the threshold effect (concentration of the air pollutant corresponding to minimum preterm birth) of NO_2_, SO_2_, and CO on preterm birth. Increased risks of preterm birth were associated with increases in the mean concentrations of NO_2_, SO_2_ and CO above thresholds in 24 or 48 hr before birth. The effect of each pollutant appeared independent of the others. We did not discover effect modification with maternal age or maternal health history. The associations of air pollutants with preterm birth risk were stronger among smokers and women from areas with relatively lower SES, and the risks associated with NO_2_ and CO were slightly greater among previously pregnant women, multiple births, and female babies.

The shape of the exposure–response curve is a critical issue in air pollution research. A threshold value of air pollutant effect is usually expected to protect population health by keeping the pollutant below this level. Regarding the adverse effects of air pollution on birth outcomes, a recent analysis (Fleischer et al. 2014) of the World Health Organization Global Survey on Maternal and Perinatal Health suggested a possible threshold effect for preterm birth in China, with a threshold of 36.5 mg/m^3^ for PM_2.5_. A Spanish study (Llop et al. 2010) observed that perinatal exposure to traffic-related air pollution above certain concentration levels was associated with preterm birth, with a threshold of 46.2 mg/m^3^ for NO_2_ and a threshold of 2.7 mg/m^3^ for benzene. In our study, we again identified evidence for threshold effect of air pollution on preterm birth. However, our findings are related to short-term effects of air pollution on preterm birth, rather than long-term effects.

Our findings of increased preterm birth associated with increases in NO_2_, SO_2_, and CO concentrations in the immediate few hours preceding onset of labor are consistent with results of previous studies that reported the short-term effects of air pollution during pregnancy on preterm birth (Leem et al. 2006; Liu et al. 2003; Zhao et al. 2011). Liu et al. (2003) found that exposure to increased SO_2_ and CO during the last month of pregnancy contributed to higher risk of preterm birth in Vancouver, Canada. Leem et al. (2006) detected dose-dependent relationships between preterm birth and exposure to NO_2_, SO_2_, and CO particularly during the third trimester of pregnancy in the Republic of Korea. A study (Zhao et al. 2011) conducted in Guangzhou, China, using a time-series design, also indicated positive associations between preterm birth and daily concentrations of NO_2_ and SO_2_. Importantly, the concentrations of air pollutants in our study were lower than those of above studies. This means that even for people living in an environment with a very low concentration of air pollution, the air pollution still has impacts on human health. Some studies also found hazard effects of daily air pollution on daily preterm birth in U.S. cities which had slightly higher concentrations of air pollutants than our study area (Darrow et al. 2009; Sagiv et al. 2005).

The very acute association between air pollution and the risk of preterm birth may suggest that ambient air pollutants can rapidly motivate the biologic mechanism of labor, leading to preterm birth in the next few hours. The potential mechanism responsible for this association may work through a series of causes including oxidative stress, inflammation, endothelial dysfunction, endocrine disruption, and hemodynamic responses (Cunningham et al. 2014; Slama et al. 2008). When pollutants are inhaled into the body, cytokines trigger oxidative stress, which could induce endothelial dysfunction to develop preeclampsia (pregnancy hypertension) (Wu et al. 2009; Yorifuji et al. 2015). Simultaneously, air pollutants cause intrauterine inflammation which increases prostaglandin levels to induce preterm premature rupture of membranes (PPROM) (Aagaard-Tillery et al. 2005; Leem et al. 2006). Preterm birth is a consequence of preeclampsia and PPROM.

Interestingly, we did not find a significant impact of PM and O_3_ on preterm birth in this study. A previous study conducted in the same study area reported that a long-term exposure to PM_10_ and O_3_ during the first trimester was associated with an increased risk of preterm birth, with OR = 1.15 (95% CI: 1.06, 1.25) and OR = 1.26 (95% CI: 1.10, 1.45), respectively. This indicates that PM and O_3_ might not have short-term effects on preterm birth, but have long-term (cumulative) effects (Hansen et al. 2006). Some studies using daily time-series air pollution and preterm birth also found nonsignificant short-term impacts of PM (Darrow et al. 2009; Sagiv et al. 2005), but reported significant impacts of NO_2_ and SO_2_ (Olsson et al. 2012; Sagiv et al. 2005). Further studies are still needed to explore why PM and O_3_ do not have short-term impacts on preterm birth.

Smoking and adverse socioeconomic characteristics are confirmed risk factors of preterm birth (Ahern et al. 2003; Ponce et al. 2005). Our findings of higher risk of preterm birth among mothers who were smoking and who lived at low-SES areas provide further evidence for the role of smoking and socioeconomic inequity in the relationship between air pollution and preterm birth. It has been reported that multiple pregnancies and experience of previous pregnancy (especially for previous experience of preterm birth) are risk factors for preterm birth (Buchmayer et al. 2004; Kurdi et al. 2004). In this study, we propose that the experience of previous pregnancy and a multiple pregnancy may aggravate the body’s response to air pollution. Therefore, multiple pregnancies and those having had a previous pregnancy should attempt to reduce the chance of exposure to air pollution to reduce the risk of preterm birth. So far, results from air pollution studies on the effect modification by sex are not uniform, and the biologic difference between boys and girls against air pollution remains unclear. A literature review summarized that male infants were generally less mature than females at term, and also at earlier gestational ages (Ghosh et al. 2007). This relatively less mature status may make them more vulnerable than females to risk factors such as air pollution. However, analysis of sex is essential in future research to elucidate this difference.

To our knowledge, this is the first study addressing the association between the very short-term (a few hours before onset of labor) air pollution exposure and the risk of preterm birth. The time-stratified case-crossover design ensured unbiased conditional logistic regression estimates and avoided time trend bias of the exposure (Janes et al. 2005). We ran one-pollutant and multiple-pollutant models to check the independent effect of the pollutant, and we tailored the data by matching on specific groups to examine the effect modification of time-invariant factors. In addition, the threshold effect of air pollution was indicated in our study, providing evidence of protection by bringing the pollutant concentration below the threshold point. A number of sensitivity analyses were performed to select the type and exposure time frame of hazardous pollutants.

Our study does have several limitations. First, we assigned mean pollutant concentrations of the specific hours preceding onset of labor to each preterm birth, ignoring pregnant women’s time spent indoors and outdoors. This may lead to exposure measurement error for the true exposure. However, we deducted the labor length when defining the time of labor onset to minimize this error. Moreover, this exposure measurement error can only underestimate the risk of preterm birth associated with increases in ambient air pollution concentrations (Zeger et al. 2000). Second, air pollution concentrations are likely to vary within and across urban study areas as a result of differences in meteorological, topographical, and environmental variables and in the type and location of emission sources. We, as well as previous studies (Dominici et al. 2006; Guo et al. 2013; Peng et al. 2009; Samet et al. 2000), used city-wide average air pollutant concentrations to assign individual exposure, which may bias effect estimates towards the null (Hutcheon et al. 2010). To improve the effect estimation, future studies should use air pollution exposure assessment tools with finer spatial resolution to characterize individual exposure such as land use regression models or interpolation methods. Third, because we did not have individual-level SES data, SES linked to the living area during pregnancy was used to assess whether low SES level had higher risks of preterm birth associated with air pollution. This might underestimate the modification effect of SES. Fourth, our analyses were confined to a citywide data set, resulting in the difficulty of generalizing the findings to other cities and other countries.

In conclusion, we found that sudden increases in the mean concentrations of ambient NO_2_, SO_2_, and CO above the threshold levels in 24 or 48 hr immediately before onset of labor stimulated preterm birth. This study provides the latest evidence that reducing air pollution to a certain level or lower could greatly benefit perinatal health, and that the influence may be quickly effective. Some maternal demographic characteristics such as smoking and socioeconomic levels may modify the air pollution effects. We have proposed some biologic mechanisms underlying these associations.

## Supplemental Material

(431 KB) PDF
