# Supplementary material for: Acute Impact of Hourly Ambient Air Pollution on Preterm Birth
Source: Environ Health Perspect. 2016 Apr 29;124(10):1623–9. doi: 10.1289/EHP200 (PMC5047774; doi:10.1289/EHP200)
Supplement: (431 KB) PDF [file EHP200.s001.acco.pdf]

Environ Health Perspect
DOI: 10.1289/EHP200

Note to readers with disabilities: *EHP* strives to ensure that all journal content is accessible to all readers. However, some figures and Supplemental Material published in *EHP* articles may not conform to 508 standards due to the complexity of the information being presented. If you need assistance accessing journal content, please contact ehp508@niehs.nih.gov. Our staff will work with you to assess and meet your accessibility needs within 3 working days.

Supplemental Material

Acute Impact of Hourly Ambient Air Pollution on Preterm Birth

Shanshan Li, Yuming Guo, and Gail Williams

Table of Contents

R codes

Table S1: Summary statistics of daily air pollution and weather conditions during 2009 – 2013 in Brisbane, Australia.

Table S2: Pearson correlation coefficients for hourly air pollution and weather conditions.

Table S3: P-values for differences for the effect estimates by level of demographic factors, corresponding to Table 5.

Figure S1: The associations between air pollutants and preterm birth by different periods of time of exposure before onset of labour.
CO: carbon monoxide; NO2: nitrogen dioxide; OR: odds ratio; PM2.5: particulate matter 2.5 micrometres or less in diameter; PM10: particulate matter 10 micrometres or less in diameter; ppb: parts per billion; SO2: sulphur dioxide; μg/m3: microgram per cubic meter.

Figure S2: The relationships between air pollutants and preterm birth in single-pollutant models with 3 degrees of freedom natural cubic splines for air pollutants. Sensitivity analyses with adjustment for temperature variability (standard deviation of 0–72 hours' temperatures), in comparison with Figure 1. CO: carbon monoxide; NO2: nitrogen dioxide; OR: odds ratio; ppb: parts per billion; SO2: sulphur dioxide.

R codes

```
############################################################
#### R codes for case-crossover design matching    ########
#### case and control by the same hour of the same ########
#### day of the week in the same month             ########
############################################################

############################################################
#### Be aware that this is simulated data. It is   ########
#### not the same as the study data                ########
############################################################



########################################################
### simulate hourly data for one year #################
########################################################
DateTime=format(seq(as.POSIXct("2009-01-01"), by=("+1 hour"),
                length.out=365*24), format="%Y-%m-%d-%H") ## date hour
date<-format(as.POSIXct(DateTime, format="%Y-%m-%d-%H"),"%Y-%m-%d") ## date
hour<-format(as.POSIXct(DateTime, format="%Y-%m-%d-%H"),"%H")  ## hour
month<-format(as.POSIXct(DateTime, format="%Y-%m-%d-%H"),"%m") ## month
dow<-format(as.POSIXct(DateTime, format="%Y-%m-%d-%H"),"%w")   ## day of the week
ymdowhour<-format(as.POSIXct(DateTime, format="%Y-%m-%d-%H"),
                  "%Y-%m-%w-%H") ## year-month-dow-hour


###### create a data set for air pollution
data<-data.frame(DateTime,date,hour,month,dow,ymdowhour)
data$NO2<-rnorm(nrow(data),mean=10,sd=5) ## create NO2
library(tsModel)  ## package for creating moving average of air pollution
data$NO2_lag0_24<-runMean(data$NO2,0:24) ## 0-24 hours' average of NO2

###### create a data set for preterm birth (PB)
###### not all hours have PB
data.PB<-data[sample(nrow(data),1000),]  ## sample 1000 hours which have preterm
birth
data.PB$sex<-sample(c("male","female"),1000,replace=T) ## create sex variable
data.PB$smoking<-sample(c("smoke","nosmoke"),1000,replace=T) ## create smoking
variable


###### match case and control
for (i in 1:nrow(data.PB)){
ymdowh_cc<-data.PB[i,]$ymdowhour
date_case<-data.PB[i,]$date
data_cc<-subset(data,data$ymdowhour%in%ymdowh_cc)
data_cc$case<-as.numeric(data_cc$date%in%date_case)
data_cc$time<-as.numeric(!data_cc$date%in%date_case)
data_cc$strata<-i
data_cc$sex<-data.PB[i,]$sex
data_cc$smoking<-data.PB[i,]$smoking
if (i==1){final.data=data_cc}else{final.data=rbind(final.data,data_cc)}
}



############################################################
##### start to perform conditional logistic regression ####
############################################################
```

```
library(survival)

### fit linear model
model1<-coxph(Surv(time,case)~NO2_lag0_24+strata(strata),
              method = c("breslow"),data=final.data)
summary(model1)
AIC(model1)

### fit non-linear model with natural cubic spline
library(dlnm)
cb.no2<-onebasis(final.data$NO2_lag0_24,fun="ns",df=4)
model2<-coxph(Surv(time,case)~cb.no2+strata(strata),
              method = c("breslow"),data=final.data)
summary(model2)
AIC(model2)
pred.no2<-crosspred(cb.no2,model2)
plot(pred.no2)
```

Table S1: Summary statistics of daily air pollution and weather conditions during 2009 – 2013 in Brisbane, Australia.

Variables	Mean ± SD	Percentiles				
		5th	25th	50th	75th	95th
PM2.5 (µg/m3)	6.32 ± 3.88	3.28	4.41	5.59	7.23	10.93
PM10 (µg/m3)	17.27 ± 14.23	9.96	13.18	15.76	18.92	25.94
NO2 (ppb)	6.52 ± 2.57	2.93	4.55	6.06	8.34	11.25
SO2 (ppb)	1.95 ± 1.38	0.71	1.15	1.53	2.18	4.77
O3 (ppb)	17.27 ± 4.56	10.26	14.06	16.99	20.1	25.12
CO (ppb)	219.25 ± 115.08	62.55	125.83	200.00	300.78	417.03
Temperature (˚C)	21.97 ± 3.69	15.67	18.92	22.75	25.11	26.94
Relative humidity (%)	70.82 ± 8.50	55.43	66.26	71.18	76.24	83.94

CO: carbon monoxide; NO2: nitrogen dioxide; O3: ozone; PM2.5: particulate matter 2.5 micrometres or less in diameter; PM10: particulate matter 10 micrometres or less in diameter; ppb: parts per billion; SD: standard division; SO2: sulphur dioxide; µg/m3: microgram per cubic meter.

Table S2: Pearson correlation coefficients for hourly air pollution and weather conditions.

	PM2.5	PM10	NO2	SO2	O3	CO	Temperature	Humidity
PM2.5	1.00							
PM10	0.71**	1.00						
NO2	0.20**	0.02**	1.00					
SO2	0.03**	0.01*	-0.03**	1.00				
O3	0.19**	0.12**	-0.34**	0.18**	1.00			
CO	0.18**	0.02**	0.43**	0.02**	-0.12**	1.00		
Temperature	-0.07**	0.04**	-0.57**	0.18**	0.45**	-0.22**	1.00	
Humidity	-0.16**	-0.17**	0.18**	-0.13**	-0.73**	0.10**	-0.37**	1.00

*p < 0.05; **p < 0.01.

CO: carbon monoxide; NO2: nitrogen dioxide; O3: ozone; PM2.5: particulate matter 2.5 micrometres or less in diameter; PM10: particulate matter 10 micrometres or less in diameter; SO2: sulphur dioxide.

Table S3: P-values for differences for the effect estimates by level of demographic factors, corresponding to Table 5.

Factors		p-value for difference		
		NO2	SO2	CO
Maternal age (years)				
< 35 VS ≥ 35	75th VS Threshold	0.69	0.61	0.79
	95th VS Threshold	0.30	0.69	0.31
Pre-pregnancy medical conditions				
No VS Yes	75th VS Threshold	0.03	0.69	0.98
	95th VS Threshold	0.05	0.52	0.78
Previous pregnancy				
0 VS ≥ 1	75th VS Threshold	0.53	0.13	0.13
	95th VS Threshold	0.07	0.79	0.19
Smoking				
Yes VS No	75th VS Threshold	0.04	0.52	0.27
	95th VS Threshold	0.04	0.42	0.24
Number of births				
Single VS Multiple	75th VS Threshold	0.38	0.15	0.84
	95th VS Threshold	0.08	0.02	0.33
Baby gender				
Female VS Male	75th VS Threshold	0.65	0.13	0.31
	95th VS Threshold	0.61	0.76	0.36
Social-economic level				
Index 1 – 5 VS	75th VS Threshold	0.64	0.76	<0.0001
Index 6 – 10	95th VS Threshold	0.94	0.32	0.002

CO: carbon monoxide; NO2: nitrogen dioxide; SO2: sulphur dioxide.

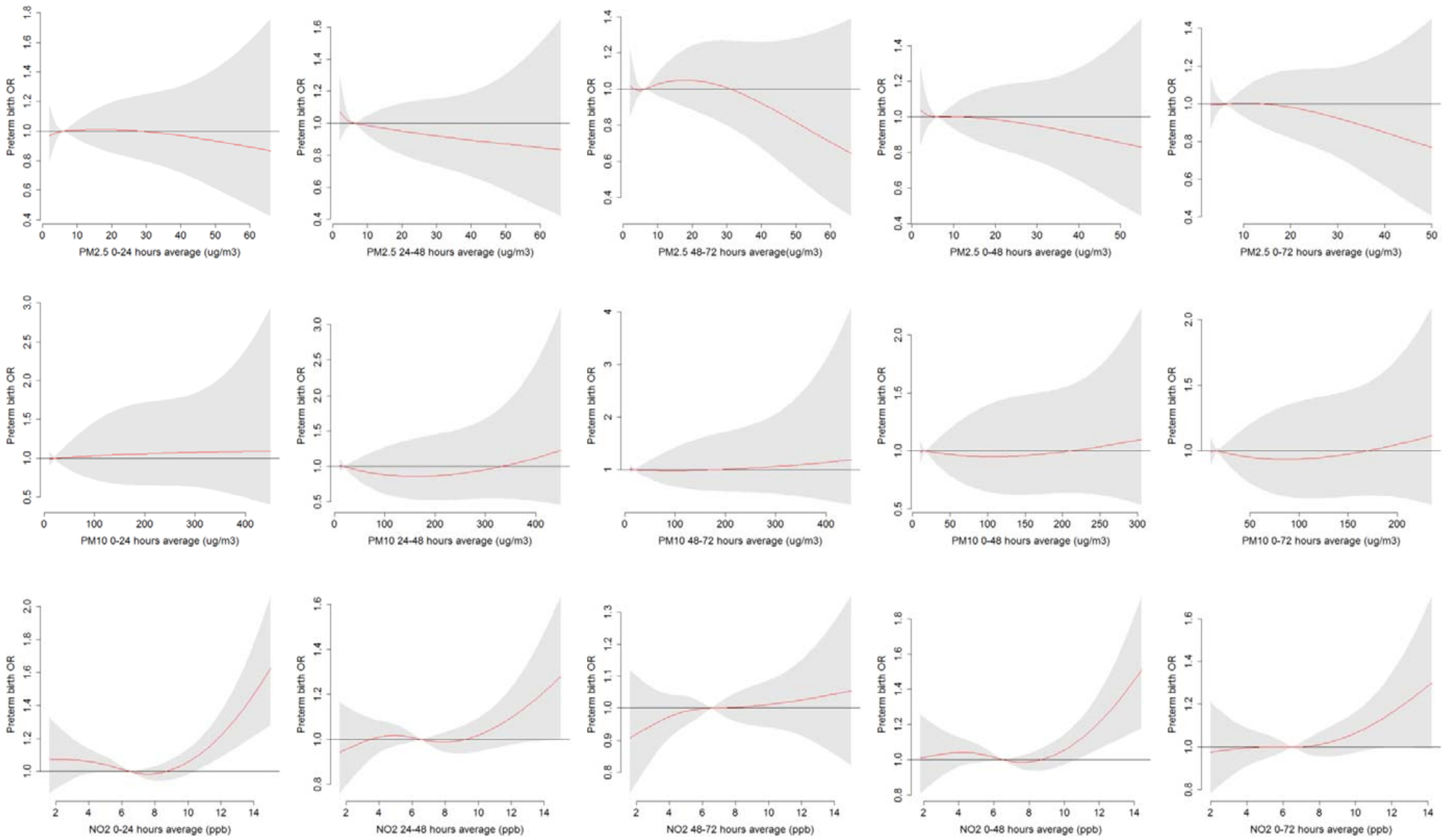
Preterm birth OR
PM2.5 0-24 hours average (ug/m3)
PM2.5 24-48 hours average (ug/m3)
PM2.5 48-72 hours average(ug/m3)
PM2.5 0-48 hours average (ug/m3)
PM2.5 0-72 hours average (ug/m3)
PM10 0-24 hours average (ug/m3)
PM10 24-48 hours average (ug/m3)
PM10 48-72 hours average (ug/m3)
PM10 0-48 hours average (ug/m3)
PM10 0-72 hours average (ug/m3)
NO2 0-24 hours average (ppb)
NO2 24-48 hours average (ppb)
NO2 48-72 hours average (ppb)
NO2 0-48 hours average (ppb)
NO2 0-72 hours average (ppb)

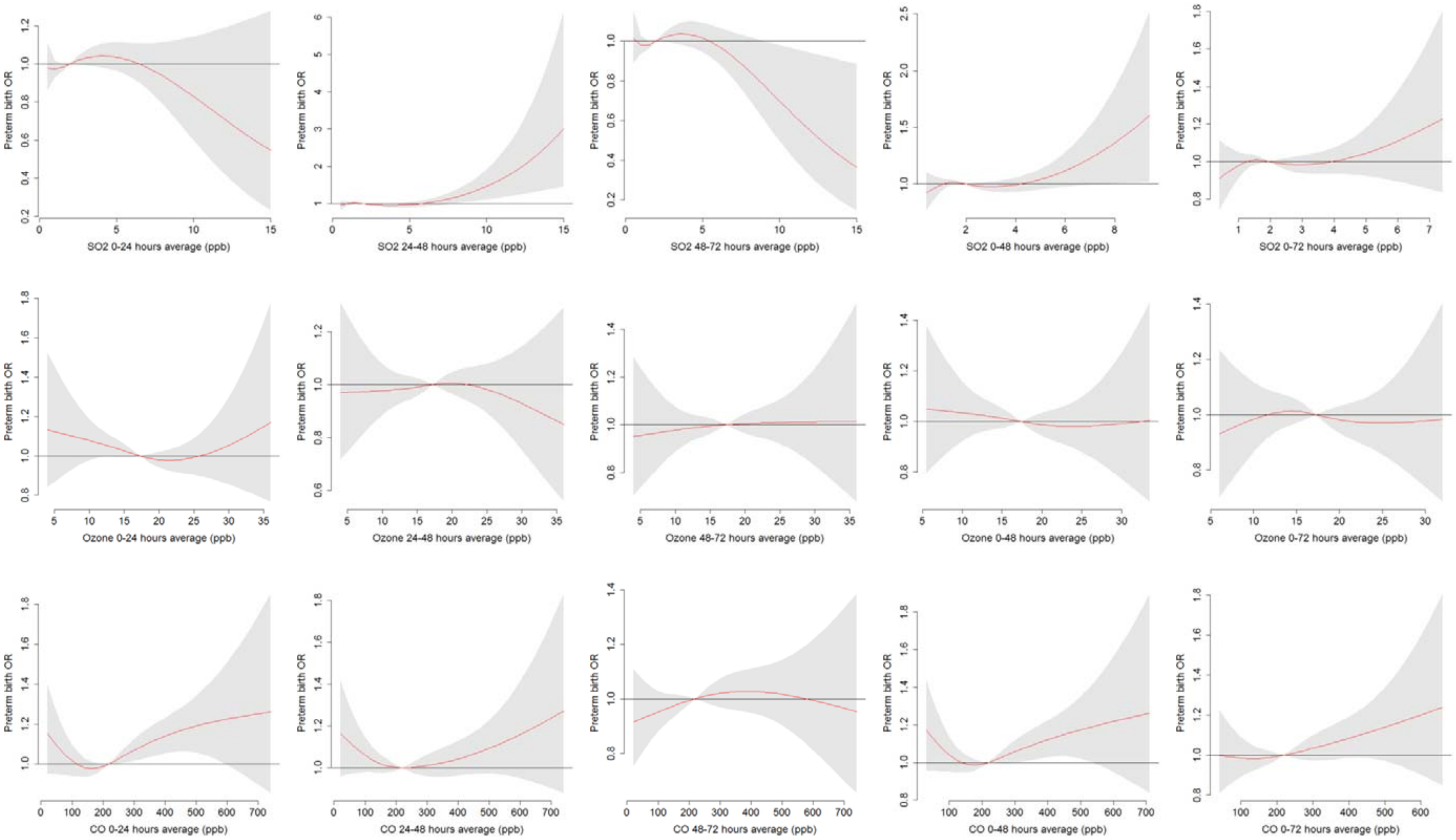

Figure S1: The associations between air pollutants and preterm birth by different periods of time of exposure before onset of labour.
CO: carbon monoxide; NO2: nitrogen dioxide; OR: odds ratio; PM2.5: particulate matter 2.5 micrometres or less in diameter; PM10: particulate matter 10 micrometres or less in diameter; ppb**:** parts per billion; SO2: sulphur dioxide; µg/m3: microgram per cubic meter.

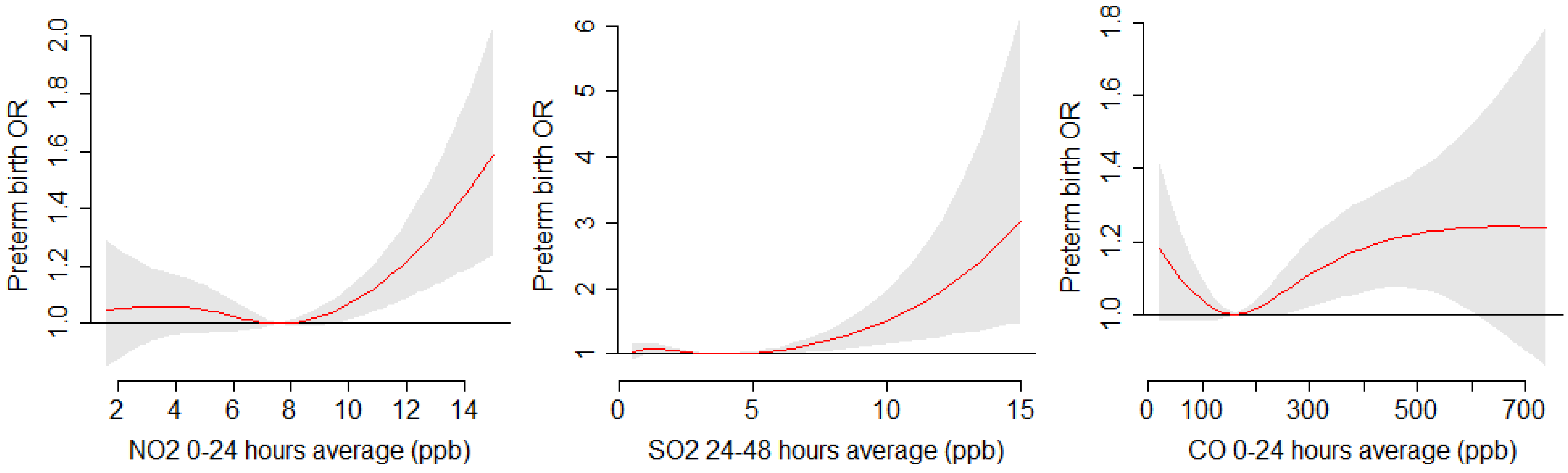

Figure S2: The relationships between air pollutants and preterm birth in single-pollutant models with 3 degrees of freedom natural cubic splines for air pollutants. Sensitivity analyses with adjustment for temperature variability (standard deviation of 0–72 hours' temperatures), in comparison with Figure 1. CO: carbon monoxide; NO2: nitrogen dioxide; OR: odds ratio; ppb: parts per billion; SO2: sulphur dioxide.
